# Supplementary material for: Predictive biomarkers for survival benefit with ramucirumab in urothelial cancer in the RANGE trial
Source: Nat Commun. 2022 Apr 6;13:1878. doi: 10.1038/s41467-022-29441-y (PMC8987042; doi:10.1038/s41467-022-29441-y)
Supplement: Supplementary file 1 — Supplementary Information [file 41467_2022_29441_MOESM1_ESM.pdf]

SUPPLEMENTARY FIGURES AND TABLES

Predictive biomarkers for survival benefit with ramucirumab in urothelial cancer: analysis of the RANGE phase 3 trial

Michiel S. van der Heijden*, Thomas Powles, Daniel Petrylak, Ronald de Wit, Andrea Necchi, Cora N. Sternberg, Nobuaki Matsubara, Hiroyuki Nishiyama, Daniel Castellano, Syed A. Hussain, Aristotelis Bamias, Georgios Gakis, Jae-Lyun Lee, Scott T. Tagawa, Ulka Vaishampayan, Jeanny B. Aragon-Ching, Bernie J. Eigl, Rebecca R. Hozak, Erik R. Rasmussen, Meng Summer Xia, Ryan Rhodes, Sameera Wijayawardana, Katherine M. Bell-McGuinn, Amit Aggarwal, Alexandra Drakaki

*Corresponding Author

Supplementary Table 1. Benjamini-Hochberg-adjusted p-values for hypothesis of superior treatment effect from ramucirumab – corresponding to comparisons in Figures 1a, 1f, 2d-e, and 3f

Subgroup		Stratified p-value without BH-adjustment	Rank of stratified p-value	BH-adjusted p-value
CPS <10		0.725	40	0.852
CPS ≥10		0.002	1.5	0.047
TC <1		0.524	32	0.770
TC ≥1		0.094	12	0.347
IC <1		0.545	33	0.776
IC ≥1		0.096	13	0.347
≤Median	Mean of immune signatures	0.848	44	0.906
	T-Effector[1,2]	0.797	42	0.892
	T-Cell Inflamed[3]	0.819	43	0.895
	Activated CD4[4]	0.685	39	0.826
	Activated CD8[4]	0.886	45	0.925
	Memory CD4[4]	0.492	29	0.760
	Memory CD8[4]	0.746	41	0.855
	Mean of angiogenesis signatures	0.237	22.5	0.484
	Liberzon[5]	0.426	28	0.715
	Brauer[6]	0.268	25	0.504
	Masiero[7]	0.208	20	0.484
	Powles[8]	0.167	17	0.440
	Uhlik_1[9]	0.501	31	0.760
	Uhlik_2[9]	0.664	38	0.821
	Uhlik_3[9]	0.178	19	0.440
>Median	Mean of immune signatures	0.005	3	0.056
	T-Effector[1,2]	0.006	4.5	0.056
	T-Cell Inflamed[3]	0.006	4.5	0.056
	Activated CD4[4]	0.002	1.5	0.047
	Activated CD8[4]	0.020	7	0.134
	Memory CD4[4]	0.069	11	0.295
	Memory CD8[4]	0.038	9	0.198
	Mean of angiogenesis signatures	0.417	27	0.715
	Liberzon[5]	0.110	14	0.369
	Brauer[6]	0.151	16	0.440
	Masiero[7]	0.561	34	0.776
	Powles[8]	0.230	21	0.484
	Uhlik_1[9]	0.175	18	0.440
	Uhlik_2[9]	0.127	15	0.398
	Uhlik_3[9]	0.651	37	0.821
Decipher	Basal	0.237	22.5	0.484
	Claudin Low	0.048	10	0.226
	Luminal	0.495	30	0.760
	Luminal Infiltrated	0.924	46	0.930
Consensus	Basal/Squamous	0.018	6	0.134
	Stroma-rich	0.605	35	0.812
	Luminal Non-Specified	0.930	47	0.930
	Luminal Papillary	0.629	36	0.821
	Luminal Unstable	0.331	26	0.598
East Asia		0.256	24	0.501
Other Region		0.034	8	0.198

Abbreviations: BH, Benjamini-Hochberg; CPS, combined positive score; IC, immune cell; TC, tumor cell.

Supplementary Table 2. Stratified overall survival hazard ratios (treatment effect) of mean and individual signature scores for immune and angiogenesis pathways, and p-values

	≤Median (n=197)			>Median (n=197)		
Biomarker signature scores	Stratified HR	95% CI	Stratified p-value	Stratified HR	95% CI	Stratified p-value
Mean of immune signatures	1.034	0.735-1.455	0.848	0.616	0.439-0.864	0.005
T-Effector[1,2]	1.045	0.749-1.457	0.797	0.613	0.432-0.870	0.006
T-Cell Inflamed[3]	1.039	0.746-1.448	0.819	0.609	0.429-0.864	0.006
Activated CD4[4]	1.073	0.763-1.509	0.685	0.574	0.406-0.811	0.002
Activated CD8[4]	0.975	0.693-1.374	0.886	0.669	0.477-0.939	0.020
Memory CD4[4]	0.881	0.614-1.264	0.492	0.735	0.527-1.024	0.069
Memory CD8[4]	0.944	0.666-1.338	0.746	0.699	0.499-0.980	0.038
Mean of angiogenesis signatures	0.803	0.558-1.156	0.237	0.876	0.635-1.207	0.417
Liberzon[5]	0.865	0.606-1.235	0.426	0.766	0.553-1.062	0.110
Brauer[6]	0.815	0.567-1.170	0.268	0.788	0.570-1.091	0.151
Masiero[7]	0.795	0.557-1.136	0.208	0.907	0.653-1.260	0.561
Powles[8]	0.777	0.543-1.112	0.167	0.809	0.572-1.144	0.230
Uhlik_1[9]	0.884	0.619-1.264	0.501	0.801	0.580-1.104	0.175
Uhlik_2[9]	0.923	0.642-1.327	0.664	0.777	0.561-1.074	0.127
Uhlik_3[9]	0.780	0.543-1.120	0.178	0.926	0.662-1.293	0.651

Abbreviations: CI, confidence interval; HR, hazard ratio.

P-values are based on two-sided Wald test and are shown before BH-adjustment. P-values after BH-adjustment are shown in Supplemental Table 1.

Supplementary Table 3. Relationship between ConsensusMIBC[a] and Decipher GSCv1[b] and TCGA 2017[c] UC molecular subtypes in the TR2 population (n=394)

	ConsensusMIBC[a]					
	**Luminal Papillary** n=97 (24.62%)	**Luminal Non-Specified** n=39 (9.9%)	**Luminal Unstable** n=61 (15.48%)	**Stroma-rich** n=75 (19.04%)	**Basal/Squamous** n=114 (28.93%)	**Neuroendocrine-like** n=8 (2.03%)
**Decipher GSCv1[b]**						
**Luminal** n=131 (33.25%)	**75**	**9**	**43**	**0**	**3**	**1**
**Luminal Infiltrated** n=55 (13.96%)	**11**	**26**	**9**	**8**	**1**	**0**
**Basal** n=150 (38.07%)	**11**	**4**	**8**	**60**	**60**	**7**
**Claudin Low** N=58 (14.72%)	**0**	**0**	**1**	**7**	**50**	**0**
**TCGA 2017[c]**						
**Luminal Papillary** n=95 (24.11%)	**67**	**2**	**24**	**0**	**2**	**0**
**Luminal** n=59 (14.97%)	**15**	**20**	**22**	**1**	**1**	**0**
**Luminal Infiltrated** n=117 (29.70%)	**14**	**17**	**10**	**65**	**11**	**0**
**Basal Squamous** n=108 (27.41%)	**0**	**0**	**2**	**7**	**98**	**1**
**Neuronal** n=15 (3.81%)	**1**	**0**	**3**	**2**	**2**	**7**

Supplementary Table 3 shows the UC molecular subtype class prevalence and percent of total for three UC classification systems in the TR2 population (N=394). [a]The ConsensusMIBC classification further refines the Decipher GSCv1 Basal subtype, splitting equally between Basal/Squamous and Stroma-rich groups. [b]The Decipher GSCv1 Claudin Low subtype shows substantial overlap with the ConsensusMIBC Basal/Squamous subtype. [c]TCGA 2017 Luminal Infiltrated is classified as ConsensusMIBC Stroma-rich in many instances.

Supplementary Table 4. Multivariate Cox models of overall survival for (a) Decipher and (b) ConsensusMIBC subtypes, stratified by geographical region, baseline ECOG PS, and visceral metastases

a Decipher subtypes model with stepwise selection from variables: mean of immune signature (>median vs ≤median), mean of angiogenesis signature (>median vs ≤median), Decipher GSCv1 subtypes (Basal Claudin-Low vs others), and their interactions with treatment arm (ramucirumab vs placebo)

Selected Variables	Cox Regression Coefficient	Standard Error	p-value
Ramucirumab	0.065	0.172	0.706
Mean of immune signature score >median	0.270	0.174	0.122
Mean of angiogenesis signature score >median	0.263	0.132	0.046*
Interaction: ramucirumab and mean of immune signature score >median	-0.476	0.239	0.047*

N=394 participants. P-values are based on two-sided Wald test without multiplicity adjustment. *Indicates p-value <0.05.
The global proportional hazard assumption was violated with p=0.02.

b ConsensusMIBC subtypes model with stepwise selection from variables: mean of immune signature (>median vs ≤median), mean of angiogenesis signature (>median vs ≤median), ConsensusMIBC subtypes (Basal/Squamous vs others) and their interactions with treatment arm (ramucirumab vs placebo)

Selected Variables	Cox Regression Coefficient	Standard Error	p-value
Ramucirumab	-0.020	0.142	0.886
Mean of angiogenesis signature score >median	0.281	0.124	0.024*
ConsensusMIBC subtype of Basal/Squamous	0.327	0.188	0.082
Interaction: ramucirumab and ConsensusMIBC subtype of Basal/Squamous	-0.568	0.269	0.035*

N=394 participants. P-values are based on two-sided Wald test without multiplicity adjustment. *Indicates p-value <0.05.
The global proportional hazard assumption was not violated.

Supplementary Table 5. Association of mean angiogenesis and immune signatures with clinical covariates, full multivariate model

	Mean of Angiogenesis Signatures		Mean of Immune Signatures	
	Linear Regression Coefficient	p-value	Linear Regression Coefficient	p-value
Gender, M vs F	-0.019	0.222	-0.027	0.126
Region, East Asia vs others	-0.063	<0.0001	-0.045	0.011
Age, 10 year-increase	-0.008	0.183	0.004	0.612
Histology, pure trans vs mixed	-0.005	0.739	-0.042	0.013
PRMSITE, BLA vs others	0.014	0.262	-0.026	0.087
VISMET, Yes vs No	0.040	0.005	-0.014	0.405
LIVMET, Yes vs No	-0.018	0.286	-0.009	0.643
Bellmunt, 1 point-increase	0.013	0.145	0.012	0.252
Prior therapy, Neo vs Adj	0.009	0.643	0.006	0.784
Prior therapy, None vs Adj	-0.004	0.770	-0.003	0.833

Abbreviations: Adj, adjuvant; BLA, bladder; Bellmunt, Bellmunt risk factors; LIVMET, liver metastases; Neo, neoadjuvant; PRMSITE, primary site; trans, transitional; VISMET, visceral metastases.

Supplementary Table 6. List of investigators and study sites, ordered alphabetically by country and investigator last name

Investigator Last Name, Degree	Investigator First, Middle Name	Site Address
Australia		
Clay, MD	Timothy Dudley	St John of God Hospital –Subiaco<br>12 Salvado Road,<br>Subiaco, WA, 6008<br>Australia
Hovey, MD	Elizabeth Jane	Prince Of Wales Hospital<br>Barker Street,<br>Randwick, NSW, 2031<br>Australia
Tan, MD	Thean Hsiang	Royal Adelaide Hospital<br>Level 6 E 351<br>Port Road Adelaide, SA, 5000<br>Australia
Wan Ng, MBBS	Siobhan Su	St John of God Hospital –Subiaco<br>12 Salvado Road,<br>Subiaco, WA, 6008<br>Australia
Wong, MD	Suet-Lai Shirley	Sunshine Hospital<br>176 Furlong Rd<br>St Albans 3021<br>Australia
Belgium		
Dumez, MD	Herlinde	Universitaire Ziekenhuizen Leuven - Campus Gasthuisberg<br>Herestraat 49<br>Leuven, 3000<br>Belgium
Machiels, MD	Jean-Pascal	Cliniques Universitaires Saint-Luc<br>Avenue Hippocrate, 10<br>Uro-oncologie (étage -1 Route 389)<br>Brussels,1200<br>Belgium
Rutten, MD	Annemie	Algemeen Ziekenhuis St Augustinus-St. Camillus-St. Bavo<br>Oosterveldlaan 24<br>Wilrijk, 2610<br>Belgium
Canada		
Cheng, MD	Susanna Yee-Shan	Centre- Odette Cancer Ctr. T2-034<br>2075 Bayview Avenue<br>Toronto, Ontario, M4N 3M5<br>Canada
Ferrario, MD	Cristiano	Jewish General Hospital<br>3755 Chemin Cote Ste-catherine<br>Pavilion E<br>Montreal, Quebec, H3T 1E2<br>Canada
Denmark		
Jensen, MD	Niels Viggo	Odense Universitetshospital<br>Sdr. Boulevard 29<br>Odense C, SYD, 5000<br>Denmark
Sengeloev, MD	Lisa	Herlev Hospital<br>Herlev Ringvej 75<br>Onkologisk Afdeling 54 B1<br>Herlev, 2730<br>Denmark
France		
Becht, MD	Catherine	Centre de cancérologie du Grand Montpellier<br>25 Rue de Clémentville<br>34070 Montpellier<br>France
Culine, MD	Stéphane	Hôpital Saint-Louis<br>1, Avenue Claude Vellefaux<br>75475 Paris Cedex 10<br>France
Joly, MD	Florence	Centre Francois Baclesse<br>3 Avenue General Harris,<br>14076 Caen Cedex 5<br>France
Laguerre, MD	Brigitte	Centre Eugene Marquis<br>Avenue Bataille Flandres-Dunkerque<br>35062 Rennes Cedex<br>France
Thibault, MD	Constance	Hôpital Européen Georges Pompidou<br>20-40 Rue Leblanc<br>75015 Paris

		France
Germany		
Grimm, MD	Marc-Oliver	Klinikum Der Friedrich-Schiller-Universität Jena<br>Am Klinikum 1, Haus A<br>Jena, THÜRINGEN, 07747<br>Germany
Niegisch, MD	Günter	Universitätsklinikum Düsseldorf<br>Moorenstraße 5<br>Düsseldorf, NORDRHEIN-WESTFALEN, 40225<br>Germany
Schultze-Seemann, MD	Wolfgang	Universitätsklinikum Freiburg<br>Hugstetter Straße 55<br>Klinik Für Urologie<br>Freiburg Im Breisgau, BADEN-WÜRTTEMBERG, 79106<br>Germany
Schwentner, MD	Christina A	KlinikumDer Eberhard-Karls- Universität Tübingen<br>Hoppe-Seyler-Straße 3<br>Tübingen, BADEN-WÜRTTEMBERG, 72076<br>Germany
Stöckle, MD	Michael	Universitätsklinikum Des Saarlandes<br>Kirrberger Straße 1<br>Urologische Klinik, Gebäude 6<br>Homburg, SAARLAND, 66421<br>Germany
Greece		
Kalofonos, MD	Haralambos	University General Hospital Of Patras<br>Rio<br>Patras, ACHAIA, 26504<br>Greece
Karavasilis, MD	Vasilis	General Hospital Of Thessaloniki Papageorgiou<br>Ring Road<br>N. Efkarpia, THESSALONIKI, 56403<br>Greece
Mavroudis, MD	Dimitrios	University General Hospital Of Heraklion<br>Stavrakia And Voutes<br>Heraklion, CRETE, 71110<br>Greece
Papandreou, MD	Christos	General Hospital Of Thessaloniki Papageorgiou<br>Ring Road<br>N. Efkarpia, THESSALONIKI, 56403<br>Greece
Hungary		
Révész, MD	Janos	Borsod-abauj-Zemplen Megyei Korhaz Es Egyetemi Oktato<br>Korhaz<br>Szentpeteri Kapu 72-76<br>Miskolc,BAZ MEGYE, 3526<br>Hungary
Israel		
Kejzman, MD	Daniel	Meir Medical Center<br>59 Tchernichovski St.,<br>Kfar Saba, 4428164<br>Israel
Leibowitz-Amit, MD	Raya	Chaim Sheba Medical Center<br>Tel Hashomer<br>Tel Hashomer, Ramat Gan, 5265601<br>Israel
Rosenbaum, MD	Eli	Rabin Medical Center<br>39 Jabotinski St.<br>Petach
Sarid, MD	David	Tel Aviv Sourasky Medical Center<br>6 Weizman St.,<br>Tel-Aviv Jaffa, 6423906<br>Israel
Italy		
Scagliotti, MD	Giorgio Vittorio	Azienda Ospedaliero - Universitaria S. Luigi Gonzaga<br>Regione Gonzole, 10<br>10043 Orbassano, TORINO<br>Italy
Bracarda, MD	Sergio	Ospedale San Donato<br>Via Pietro Nenni, 20<br>52100 Arezzo

		Italy<br>Massari, MD Francesco Policlinico
Massari, MD	Francesco	Policlinico S. Orsola Malpighi<br>Via Albertoni, 15 Oncologia Medica,<br>Padiglione 2 Piano 5<br>40138 Bologna<br>Italy
Japan		
Fukasawa, MD	Satoshi	Chiba Cancer Center<br>666-2 Nitona-cho Chuo-Ku<br>Chiba, CHIBA, 260-8717<br>Japan
Fukuta, MD	Fumimasa	Sapporo Medical University Hospital<br>16-291 Minami-1jyo-nishi Chuo-ku<br>Sapporo, HOKKAIDO, 060-8543<br>Japan
Hashine, MD	Katsuyoshi	National Hospital Organization Shikoku Cancer Center<br>160 Kou Minamiumemoto-Machi<br>Matsuyama, EHIME, 791-0280<br>Japan
Inokuchi, MD	Junichi	Kyushu University Hospital<br>3-1-1 Maidashi Higashi-Ku<br>Fukuoka, FUKUOKA, 812-8582<br>Japan
Kawai, MD	Koji	University of Tsukuba Hospital<br>2-1-1 Amakubo<br>Tsukuba, IBARAKI, 305-8576<br>Japan
Kawakita, MD	Mutsushi	Kobe City Medical Center General Hospital<br>2-1-1, Minami-Machi, Minatojima, Chuo-Ku<br>Kobe, HYOGO, 650-0047<br>Japan
Miyajima, MD	Naoto	Hokkaido University Hospital<br>Kita 14, Nishi 5, Kita-Ku<br>Sapporo, HOKKAIDO, 060-8648<br>Japan
Nagamori, MD	Satoshi	National Hospital Organization Hokkaido Cancer Center<br>2-3-54 Kikusui-4jyo Shiroishi-Ku<br>Sapporo, HOKKAIDO, 003-0804<br>Japan
Nagata, MD	Masayoshi	Juntendo University Hospital<br>3-1-3 Hongo<br>Bunkyo-Ku, TOKYO, 113-8431<br>Japan
Nishimura, MD	Kazuo	Osaka International Cancer Institute<br>3-1-69 Otemae Chuou-Ku<br>Osaka, OSAKA, 541-8567<br>Japan
Obara, MD	Wataru	Iwate Medical University Hospital<br>19-1, Uchimaru<br>Morioka, IWATE, 020-8505<br>Japan
Ohyama, MD	Chikara	Hirosaki University Hospital<br>53 Hon-cho<br>Hirosaki, AOMORI, 036-8563<br>Japan
Osawa, MD	Takahiro	Hokkaido University Hospital<br>Kita 14, Nishi 5, Kita-Ku<br>Sapporo, HOKKAIDO, 060-8648<br>Japan
Oyama, MD	Masafumi	Saitama Medical University International Medical Center<br>1397-1 Yamane<br>Hidaka, SAITAMA, 350-1298<br>Japan
Shinohara, MD	Nobuo	Hokkaido University Hospital<br>Kita 14, Nishi 5, Kita-Ku<br>Sapporo, HOKKAIDO, 060-8648<br>Japan
Tomita, MD	Yoshihiko	Niigata University Medical & Dental Hospital<br>1-754, Asahimachidori,Chuo-ku<br>Niigata, NIIGATA, 951-8520<br>Japan
Tsunemori, MD	Hiroyuki	Kagawa University Hospital<br>1750-1 Ikenobe, Miki-cho<br>Kita-gun, Kagawa, 761-0793<br>Japan

Uemura, MD	Motohide	Osaka University Hospital<br>2-15 Yamadaoka<br>Suita-Shi, OSAKA, 565-0871<br>Japan
Yamashita, MD	Shinichi	Tohoku University Hospital<br>1-1, Seiryo-cho Aoba-Ku<br>Sendai, MIYAGI, 980-8574<br>Japan
Yokomizo, MD	Akira	Kyushu University Hospital<br>3-1-1 Maidashi Higashi-Ku<br>Fukuoka, FUKUOKA, 812-8582<br>Japan
Yonese, MD	Junji	The Cancer Institute Hospital Of JFCR<br>3-8-31 Ariake<br>Koto-Ku, TOKYO, 135-8550<br>Japan
Korea, South		
Kim, MD	Yu Jung	Seoul National University Bundang Hospital<br>300 Gumi-dong, Beon-Gil<br>Bundang-gu<br>Seongnam-si, Gyeongg1-do, 13620<br>Korea, South
Lee, MD	Hyo Jin	Chungnam National University Hospital<br>282 Munhwa-ro, Jung-gu<br>Daejeon, 35015<br>Korea, South
Lee, MD	Yun-Gyoo	Kangbuk Samsung Hospital<br>29,Saemunan-ro,, Jongro-gu<br>Seoul, 03181<br>Korea, South
Park, MD	Se Hoon	Samsung Medical Center<br>81 Irwon-ro, Gangnam-gu<br>Seoul, 06351,<br>Korea, South
Rha, MD	Sun Young	Severance Hospital Yonsei University Health System<br>50-1 Yonsei-ro, Seodaemun-gu<br>Seoul, 03722<br>Korea, South
Mexico		
Lorena Urzua Flores, MD	Claudia	Hospital Cardiologica Aguascalientes<br>República De Ecuador No. 200, Las Americas<br>Aguascalientes, AGS, 20230<br>Mexico
Vazquez Cortés, MD	Leticia	Centro De Investigacion Clinica Chapultepec S.A. De C.v.<br>General Pena Y Pena 256 Col. Chapultepec Norte<br>Morelia, MICHOACÁN, 58260<br>Mexico
Netherlands		
Aarts, MD	Maureen J.B.	Universitair Medisch Centrum Maastricht<br>P. Debyelaan 25<br>Maastricht, 6229 HX,<br>The Netherlands
Blaisse, MD	Reinoud J.B.	Rijnstate Ziekhenius<br>Wagnerlaan 55<br>Arnhem, 6815 AD,<br>The Netherlands
Erdkamp, MD	Fransiscus L.G.	Zuyderland MC<br>Dr. H. Van Der Hoffplein 1<br>Sittard – Geleen, 6162 BG,<br>The Netherlands
Poland		
Sikora-Kupis, MD	Bozena	MAGODENT Sp. z o.o. Szpital Elbląska<br>ul. Szamocka 6,<br>01-748 Warszawa<br>Poland<br><br>And<br><br>Magodent Sp. Z O.o.<br>Ul. Gen. Fieldorfa 40<br>Warszawa, 04-125<br>Poland
Tomczak, MD	Piotr	Szpital Kliniczny Przemienienia Pańskiego<br>Uniwersytetu Medycznego im. Karola Marcinkowskiego w Poznaniu

		Oddział Chemioterapii<br>ul. Szamarzewskiego 82/84<br>60-569 Poznań<br>Poland
Wojcik-Tomaszewska, MD	Joanna	Copernicus PL Sp. z o.o. Wojewódzkie Centrum Onkologii<br>Aleja Zwycięstwa 31/32<br>80-219 Gdańsk<br>Poland
Romania		
Herzal, MD	Alina Amalia	Spitalul Judetean De Urgenta "dr. Constantin Opris"<br>Str. George Cosbuc, Nr. 31<br>Baia Mare, 430031<br>Romania
Schenker, MD	Michael	Centrul De Oncologie Sf. Nectarie SRL<br>Str. Caracal nr. 23a, Parter Si Demisol, Bloc 17a<br>Craiova, DOLJ, 200347<br>Romania
Udrea, MD	Anghel Adrian	S.C. Medisprof SRL<br>Piata 1 Mai Nr. 3<br>Cluj-Napoca, Cluj, 400058<br>Romania
Russian Federation		
Fomkin, MD	Roman	Saratov State Medical University<br>137, Bolshya sadovaya ul.<br>Saratov, 410054<br>Russian Federation
Karlov, MD	Petr	St. Petersburg City Clinical Oncological Dispensary<br>56 Veteranov Prospect<br>Saint-Petersburg, 198255<br>Russian Federation
Spain		
Delgado Mignorance	Juan Ignacio	Hospital Infanta Cristina<br>Avda. De Elvas, S/n<br>Badajoz, BADAJOZ, 06080<br>Spain
Gajate Borau, MD	Pablo	Hospital Universitario Ramon y Cajal<br>Ctra. Colmenar Viejo, Km 9.1<br>Oficina De Ensayos Clínicos/serv.onco Planta - 2 Dcha<br>Madrid, MADRID, 28034<br>Spain
Grande, MD	Enrique	Hospital Universitario Ramon y Cajal<br>Ctra. Colmenar Viejo, Km 9.1<br>Oficina De Ensayos Clínicos/serv.onco Planta - 2 Dcha<br>Madrid, MADRID, 28034<br>Spain
Taiwan		
Li, MD	Jian-Ri	Division of Urology, Department of Surgery<br>Taichung Veterans General Hospital<br>1650 Taiwan Boulevard Sect.4,<br>Taichung, Taiwan 40705, ROC
Lin, MD	Chia-Chi	National Taiwan University Hospital<br>No 1 Changde St.<br>Rm.6451,4f, 6 West Building<br>Taipei, 10048<br>Taiwan
Lin, MD	Chien-Liang	Chi-Mei Medical Center, Liouying<br>No. 201, Taikang Village, B2 5 Building<br>Tainan, 73657<br>Taiwan
Su, MD	Yu-Li	Chang Gung Memorial Hospital - Kaohsiung<br>No.123, Dapi Rd., Niaosong<br>Dist. 7f. Blood Smear Exam Rm.<br>Kaohsiung, 83301<br>Taiwan
Yeh, MD	Su-Peng	China Medical University Hospital<br>No 2, Yuh-Der Rd.,<br>Taichung, 40447<br>Taiwan
Turkey		
Erman, MD	Mustafa	Hacettepe University Faculty Of Medicine<br>Tip Fakultesi Hastanesi Sihhiye<br>Ankara, 06100

		Turkey
Urun, MD	Yuksel	Ankara University<br>Cebeci Yerleskesi<br>Ankara, 06100<br>Turkey
Ukraine		
Bondarenko, MD	Igor	Dnipropetr City Multif Cli Hosp 4 Dnip<br>Regi Council (Municipal Institution<br>"Dnipropetrovsk City Multifunctional<br>Clinical Hospital #4" of Dnipropetrovsk<br>regional council),<br>Dep Che<br>31 Blyzhnya Str<br>Dnipro, 49102, Ukraine
Golovko, MD	Yuriy	Municipal Institution «Kyiv Regional<br>Oncology Dispensary» of Kyiv regional<br>council<br>1-a, Baggovutivska Street<br>Kyiv, 04107<br>Ukraine
Sinielnikov, MD	Ivan	Health Care Institution Volyn Regional<br>Oncology Dispensary<br>1 Timiryaziva Street<br>Lutsk, 43018<br>Ukraine
United Kingdom		
Chowdhury, MD	Simon	Sarah Canon Research Institute UK Ltd<br>93 Harley Street<br>London, SURREY, W1G 6AD<br>United Kingdom
Crabb, MD	Simon J.	University Hospital Southampton NHS<br>FOUNDATION TRUST,<br>Tremona Road,<br>Southampton, SO16 6YD<br>United Kingdom
Huddart, MD	Robert	Royal Marsden NHS Foundation Trust<br>Downs Road<br>Sutton, SURREY, SM2 5PT<br>United Kingdom
Sarwar, MD	Naveed	Charing Cross Hospital<br>Fulham Palace Road<br>Chelsea, LONDON, W6 8RF<br>United Kingdom
Sundar, MD	Santhanam	Nottingham University Hospital<br>Hucknall Road Nottingham<br>NOTTINGHAMSHIRE, NG5 1PB<br>United Kingdom
Syndikus, MD	Isabel	The Clatterbridge Cancer Centre<br>NHS Foundation Trust<br>Clatterbridge Road<br>Bebington, Wirral, CH63 4JY<br>United Kingdom
United States		
Acs, MD	Peter Istvan	Florida Cancer Specialists And Research<br>Institute<br>560 Jackson Street, Suite 220<br>St Petersburg, Florida, 33705<br>United States
Cultrera, MD	Jennifer Lyn	Florida Cancer Specialists And Research<br>Institute<br>560 Jackson Street, Suite 220<br>St Petersburg, Florida, 33705<br>United States
Flaig, MD	Thomas W.	University of Colorado<br>12801 E. 17th Ave., Rc-1 South<br>Rm 8123, Ms 8117<br>Aurora, Colorado, 80045<br>United States
Hainsworth, MD	John D.	Tennessee Oncology, PLLC<br>250 25th Ave N, Suite 307<br>Nashville, Tennessee, 37203<br>United States
Herms, MD	Benjamin T.	Oncology Hematology Care Inc<br>4350 Malsbary Road<br>Cincinnati, Ohio, 45242<br>United States
Lawler, MD	William Eyre	St Jude Hospital Yorba Linda DBA<br>St. Joseph Heritage Healthcare<br>2151 N Harbor Blvd STE 2200

		Fullerton, California, 92835<br>United States
Lowe, MD	Thomas Eugene	Torrance Health Association, DBA Torrance Memorial<br>Physician Network/Cancer Care Associates<br>514 N. Prospect Ave.<br>4th Floor<br>Redondo Beach, California, 90277<br>United States
Pan, MD	Chong Xian	University Of California, Davis - Health Systems<br>4501 X Street<br>Sac ento, California, 95817<br>United States
Schwarz, MD	James K.	Fred and Pamela Buffett Cancer Center'986840<br>Nebraska Medical Center<br>Omaha, Nebraska 68198-6840<br>United States<br><br>And<br><br>University Of Nebraska Medical Center<br>987680 Nebraska Medical Center<br>Dept of Internal Medicine<br>Omaha, Nebraska, 68198-7680<br>United States

Supplementary Figure 1. Subpopulation treatment effect pattern plots for (a) PD-L1 CPS, (b) angiogenesis mean, and (c) immune mean biomarker scores

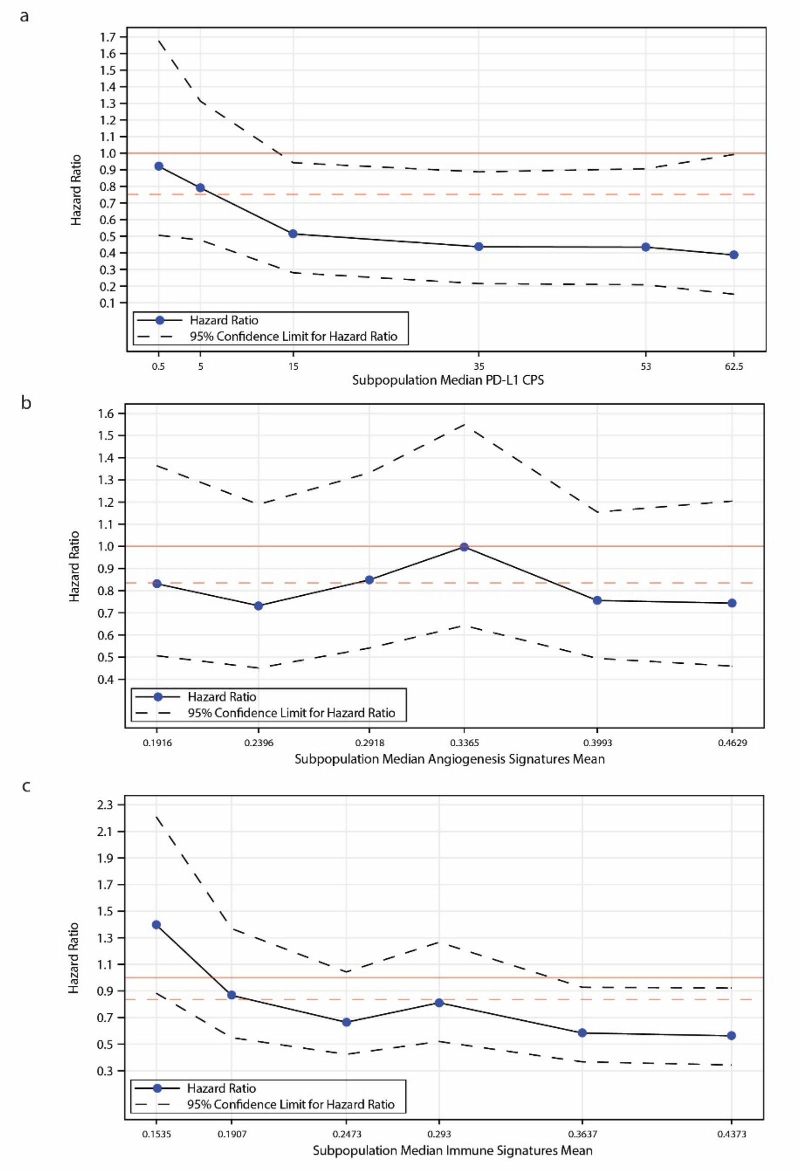

Supplementary Figure 1 Legend

Subpopulation treatment effect pattern plots showing the magnitude of the treatment effect changes as a function of the continuous measures of the biomarkers. Each dot represents the HR estimate of ramucirumab + docetaxel versus placebo + docetaxel in a sampled subpopulation with size approximately 60 patients for (**a**) and 120 patients for (**b-c**), and with median biomarker values as shown on the x-axis. Adjacent subgroups are overlapped with a maximum of 40 patients for (**a**) and 60 patients for (**b-c**). The dashed black lines represent the 95% confidence interval of hazard ratio (HR). The solid red lines represent no treatment effect (HR=1). The dashed red lines represent the HR estimate of all patients in the TR population (TR1=227 in (**a**) and TR2=394 in (**b-c**). Abbreviations: CPS, combined positive score; HR, hazard ratio; PD-L1, programmed cell death ligand; STEPP, subpopulation treatment effect pattern plots; TR, translational research.

Supplementary Figure 2. TCGA 2017 Kaplan Meier plots

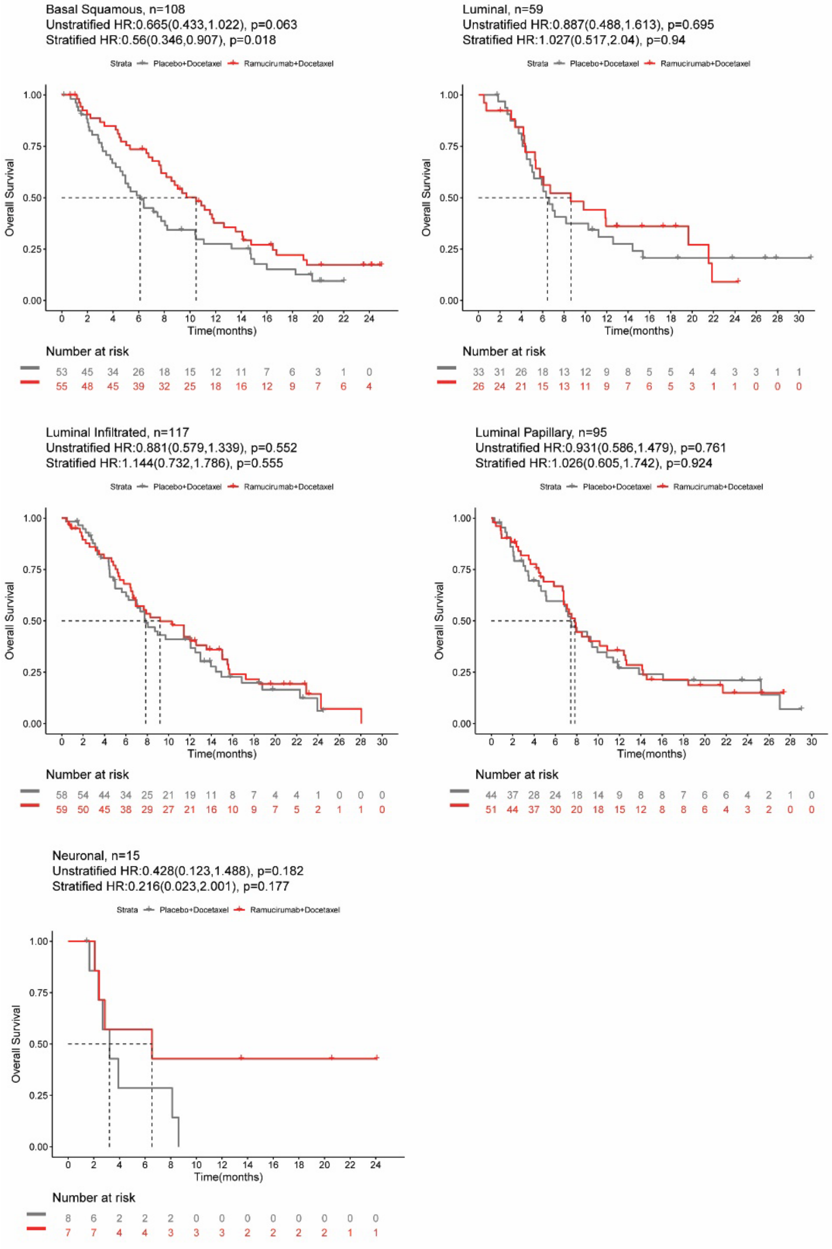

Supplementary Figure 2 Legend

Kaplan-Meier curves representing overall survival probability in ramucirumab + docetaxel or placebo + docetaxel arms based on TCGA 2017. Stratification was based on geographical region, baseline ECOG PS, and visceral metastases. **P-values are based on t**wo-sided Wald test without multiplicity adjustment. TR2 population (n=394 participants) is used. Number of participants for each subgroup is shown in the panel title. The proportional hazard assumption was not violated in any instance. Abbreviations: HR, hazard ratio; TCGA, The Cancer Genome Atlas.

Supplementary Figure 3. Association of angiogenesis and immune signatures with (a) Decipher GSCv1 and (b) ConsensusMIBC molecular subtypes

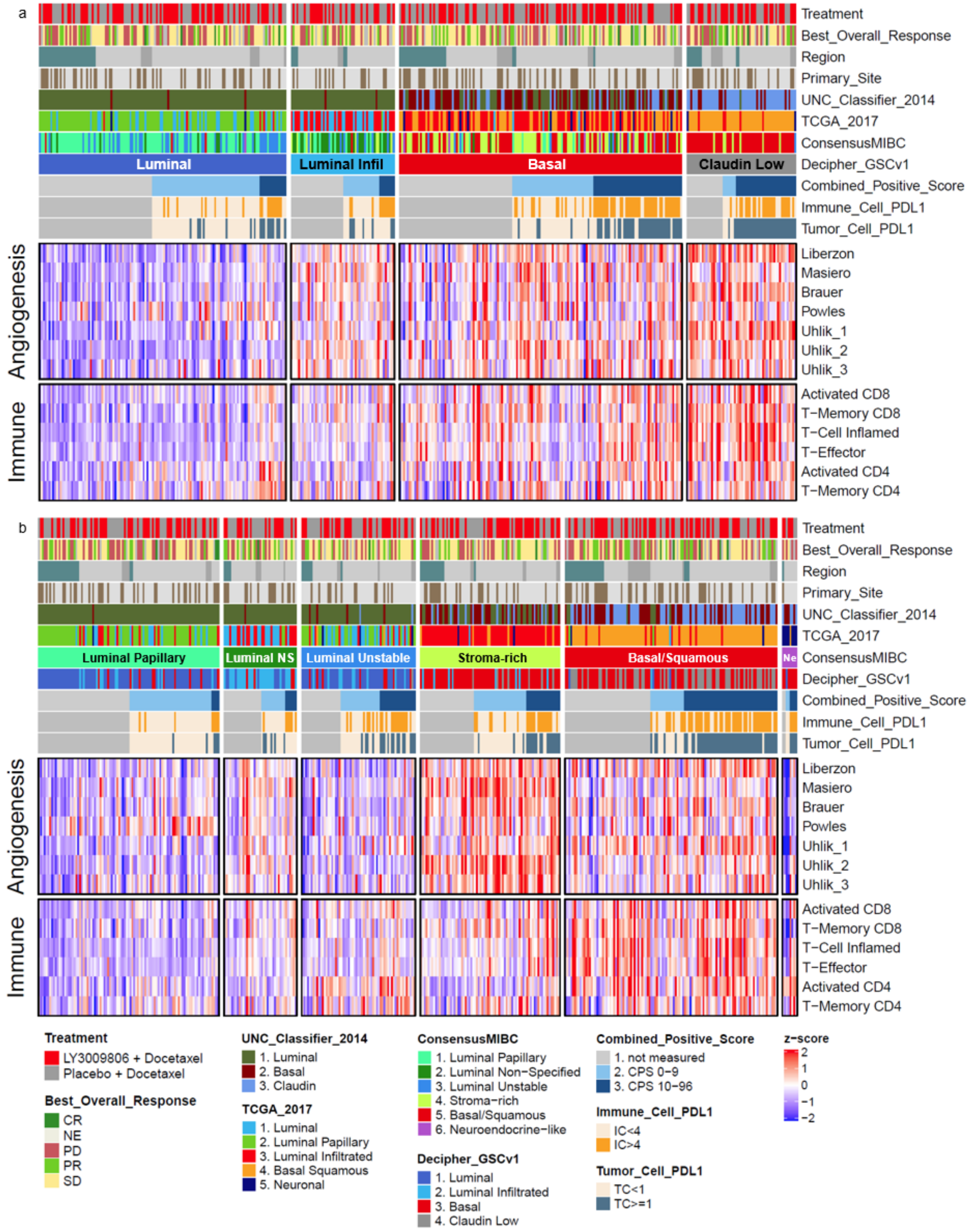

Supplementary Figure 3 Legend

Heatmaps for TR2 population (N=394) (**a**) Heatmap columns ordered by Decipher GSCv1 molecular subtype, CPS, and region. (**b**) Heatmap columns ordered by ConsensusMIBC molecular subtype, CPS, and region. Abbreviations: CPS, combined positive score; CR, complete response; IC, immune cell; Ne, neuroendocrine-like (when referring to subtype); NE, not evaluable (in Best Overall Response legend); NS, non-specified; PD, progressive disease; PD-L1, programmed cell death ligand 1; PR, partial response; SD, stable disease; TC, tumor cell.

Supplementary Figure 4. Angiogenesis and immune mean signature score in relation to visceral metastases

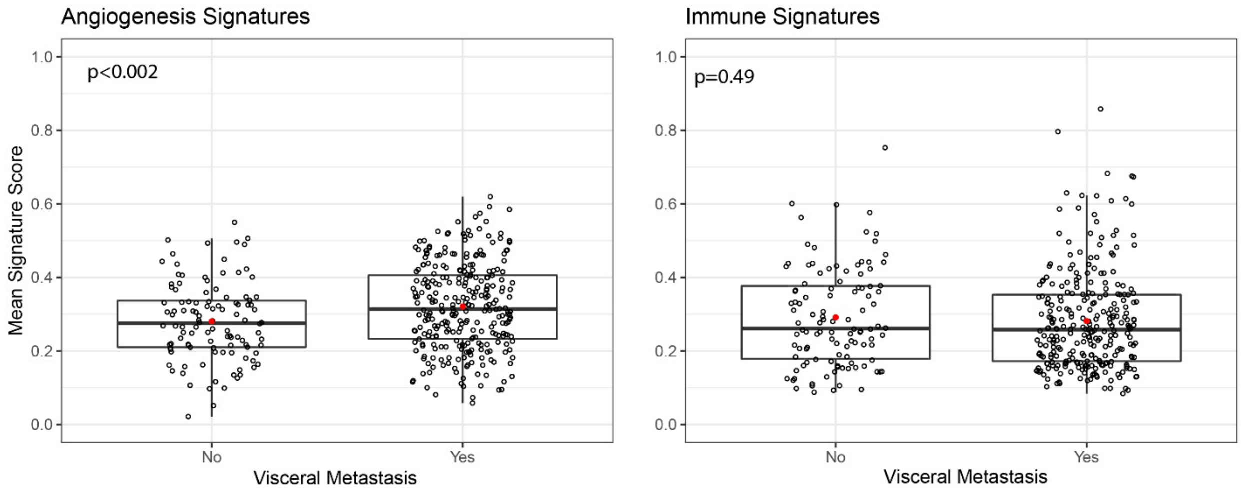

Supplementary Figure 4 Legend

Angiogenesis and immune pathway mean signature score in relation to presence of visceral metastases (No n=110, Yes n=284) in the TR2 population (n=394). For boxplots, center line represents median, box hinges represent first and third quartiles, whiskers represent minimum and maximum within 1.5x interquartile range, and red marker is mean. Mean angiogenesis signature score, $p<0.002$ (two-sample t-test not adjusted for multiplicity). Mean immune signature score, $p=0.49$ (two-sample t-test not adjusted for multiplicity).

Supplementary Figure 5. Correlation matrices showing the association between mean signature set score and individual angiogenesis and immune signatures

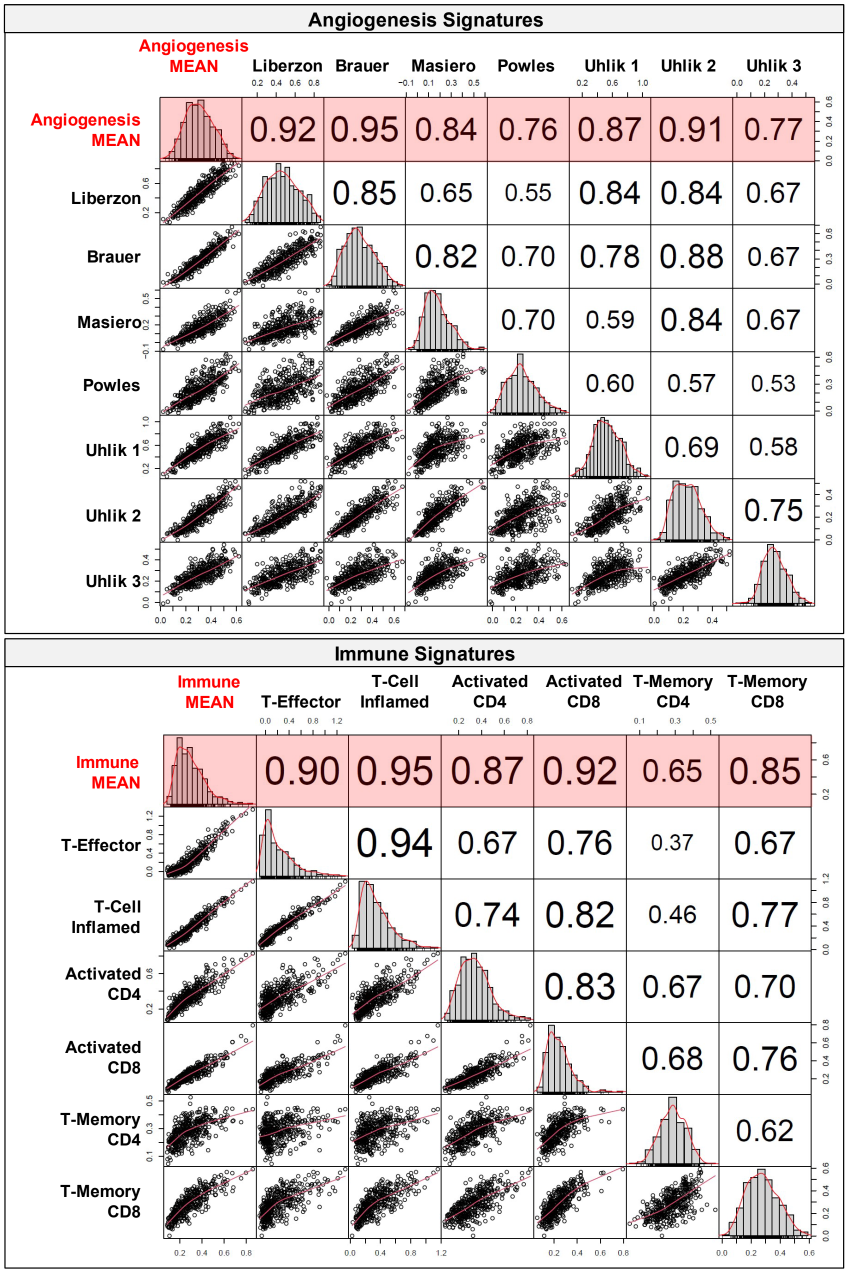

Supplementary Figure 5 Legend

Signature correlations for the TR2 population (n=394). Spearman correlation coefficients were based on log2 SCAN normalized signature scores. Within each set, all angiogenesis and immune signatures, including the average score (shaded box), showed strong positive, significant intercorrelations (p<0.001).

Supplementary References

1. Herbst, R.S., *et al.* Predictive correlates of response to the anti-PD-L1 antibody MPDL3280A in cancer patients. *Nature* **515**, 563-567 (2014).

2. Kowanetz, M., *et al.* Differential regulation of PD-L1 expression by immune and tumor cells in NSCLC and the response to treatment with atezolizumab (anti-PD-L1). *Proc. Natl. Acad. Sci. U. S. A.* **115**, E10119-E10126 (2018).

3. Ayers, M., *et al.* IFN-gamma-related mRNA profile predicts clinical response to PD-1 blockade. *J. Clin. Invest.* **127**, 2930-2940 (2017).

4. Charoentong, P., *et al.* Pan-cancer immunogenomic analyses reveal genotype-immunophenotype relationships and predictors of response to checkpoint blockade. *Cell Rep.* **18**, 248-262 (2017).

5. Liberzon, A., *et al.* The Molecular Signatures Database (MSigDB) hallmark gene set collection. *Cell Syst.* **1**, 417-425 (2015).

6. Brauer, M.J., *et al.* Identification and analysis of in vivo VEGF downstream markers link VEGF pathway activity with efficacy of anti-VEGF therapies. *Clin. Cancer Res.* **19**, 3681-3692 (2013).

7. Masiero, M., *et al.* A core human primary tumor angiogenesis signature identifies the endothelial orphan receptor ELTD1 as a key regulator of angiogenesis. *Cancer Cell.* **24**, 229-241 (2013).

8. McDermott, D.F., et al. Clinical activity and molecular correlates of response to atezolizumab alone or in combination with bevacizumab versus sunitinib in renal cell carcinoma. *Nat. Med.* **24,** 749-757 (2018).

9. Uhlik, M.T., *et al.* Stromal-based signatures for the classification of gastric cancer. *Cancer Res.* **76**, 2573-2586 (2016).
